# Cancer immunotherapy: the beginning of the end of cancer?

**DOI:** 10.1186/s12916-016-0623-5

**Published:** 2016-05-05

**Authors:** Sofia Farkona, Eleftherios P. Diamandis, Ivan M. Blasutig

**Affiliations:** Department of Laboratory Medicine and Pathobiology, University of Toronto, Toronto, ON Canada; Department of Pathology and Laboratory Medicine, Mount Sinai Hospital, Toronto, ON Canada; Department of Clinical Biochemistry, University Health Network, Toronto, ON Canada; Clinical Biochemistry, Toronto General Hospital, 200 Elizabet St. Rm 3EB-365, Toronto, ON M5G2C4 Canada

**Keywords:** Cancer, Immunotherapy, T cells, Adoptive cellular therapy, Cytotoxic T lymphocyte-associated protein 4, Programmed cell death protein 1, Immune checkpoint blockade

## Abstract

These are exciting times for cancer immunotherapy. After many years of disappointing results, the tide has finally changed and immunotherapy has become a clinically validated treatment for many cancers. Immunotherapeutic strategies include cancer vaccines, oncolytic viruses, adoptive transfer of ex vivo activated T and natural killer cells, and administration of antibodies or recombinant proteins that either costimulate cells or block the so-called immune checkpoint pathways. The recent success of several immunotherapeutic regimes, such as monoclonal antibody blocking of cytotoxic T lymphocyte-associated protein 4 (CTLA-4) and programmed cell death protein 1 (PD1), has boosted the development of this treatment modality, with the consequence that new therapeutic targets and schemes which combine various immunological agents are now being described at a breathtaking pace. In this review, we outline some of the main strategies in cancer immunotherapy (cancer vaccines, adoptive cellular immunotherapy, immune checkpoint blockade, and oncolytic viruses) and discuss the progress in the synergistic design of immune-targeting combination therapies.

## Background

The idea of exploiting the host’s immune system to treat cancer dates back decades and relies on the insight that the immune system can eliminate malignant cells during initial transformation in a process termed immune surveillance [1]. Individual human tumors arise through a combination of genetic and epigenetic changes that facilitate immortality, but at the same time create foreign antigens, the so-called neo-antigens, which should render neoplastic cells detectable by the immune system and target them for destruction. Nevertheless, although the immune system is capable of noticing differences in protein structure at the atomic level, cancer cells manage to escape immune recognition and subsequent destruction. To achieve this, tumors develop multiple resistance mechanisms, including local immune evasion, induction of tolerance, and systemic disruption of T cell signaling. Moreover, in a process termed immune editing, immune recognition of malignant cells imposes a selective pressure on developing neoplasms, resulting in the outgrowth of less immunogenic and more apoptosis-resistant neoplastic cells [2].

Scientists have known for decades that cancer cells are particularly efficient at suppressing the body’s natural immune response, which is why most treatments exploit other means, such as surgery, radiation therapy and chemotherapy, to eliminate neoplastic cells. It is now established that various components of the immune system play pivotal roles in protecting humans from cancer. Following numerous disappointing efforts and unequivocal clinical failures, the field of cancer immunotherapy has recently received a significant boost, encouraged primarily by the approval of the autologous cellular immunotherapy, sipuleucel-T, for the treatment of prostate cancer in 2010 [3] and the approval of the anti-cytotoxic T lymphocyte-associated protein 4 (CTLA-4) antibody, ipilimumab, and of anti-programmed cell death protein 1 (PD1) antibodies for the treatment of melanoma in 2011 and 2014, [4] respectively. These successes have revitalized the field and brought attention to the opportunities that immunotherapeutic approaches can offer [5].

Immunotherapies against existing cancers include various approaches, ranging from stimulating effector mechanisms to counteracting inhibitory and suppressive mechanisms (Table 1). Strategies to activate effector immune cells include vaccination with tumor antigens or augmentation of antigen presentations to increase the ability of the patient’s own immune system to mount an immune response against neoplastic cells [6]. Additional stimulatory strategies encompass adoptive cellular therapy (ACT) in an attempt to administer immune cells directly to patients, the administration of oncolytic viruses (OVs) for the initiation of systemic antitumor immunity, and the use of antibodies targeting members of the tumor necrosis factor receptor superfamily so as to supply co-stimulatory signals to enhance T cell activity. Strategies to neutralize immunosuppressor mechanisms include chemotherapy (cyclophosphamide), the use of antibodies as a means to diminish regulatory T cells (CD25-targeted antibodies), and the use of antibodies against immune-checkpoint molecules such as CTLA-4 and PD1. This review summarizes the main strategies in cancer immunotherapy and discusses recent advances in the design of synergistic combination strategies [1].

**Table 1:** The spectrum of available immunotherapies

Strategy	Basic mechanism and major advantages	Major disadvantages	Reference
Cytokines			
IL-2	-Stimulates the host’s immune system	-Low response rates -Significant risk of serious systemic inflammation	[1]
IFN-α	-Stimulates the host’s immune system -Durable responses (from a small subset of melanoma patients)	-Low response rates -High-dose toxicity	[1]
Cell-based therapies			
Vaccines	-Stimulates the host’s immune system -Minimal toxicity (e.g., sipuleucel-T) -Administered in the outpatient clinic	-Lack of universal antigens and ideal immunization protocols lead to poor efficacy and response	[6]
Adoptive cellular therapy	-Omits the task of breaking tolerance to tumor antigens -Produces a high avidity in effector T cells -Lymphodepleting conditioning regimen prior to TIL infusion enhances efficacy -Genetic T cell engineering broadens TIL to malignancies other than melanoma	-Restricted to melanoma -Safety issues, serious adverse effects, and lack of long lasting responses in many patients -Requires time to develop the desired cell populations -Expensive	[5, 27, 60, 62–64, 68–70]
Immune checkpoint blockade			
Anti-CTLA-4 monoclonal antibodies	-Unleashes pre-existing anticancer T cell responses and possibly triggers new -Exhibits potent antitumor properties -Prolongation of overall survival	-Only a relatively small fraction of patients obtain clinical benefit -Severe immune-related adverse events have been observed in up to 35 % of patients	[5, 13, 76, 77]
Anti-PD1 and anti-PD-L1 antibodies	-Sufficient clinical responses which are often long-lasting -Therapeutic responses in patients within a broad range of human cancers -Reduced toxicity compared to anti-CTLA-4 antibodies	-Only a relatively small fraction of patients obtain clinical benefit	[2, 84, 90]
Combination immunotherapy (immune checkpoint blockade as the backbone)	-Improvement of anti-tumor responses/immunity	-May lead to increases in the magnitude, frequency, and onset of side effects	[9, 10]

IL-2, Interleukin 2; IFN-α, Interferon-alpha; CTLA-4, Cytotoxic T lymphocyte-associated protein 4; PD1, Programmed cell death protein 1; TIL, Tumor infiltrating antibodies

## Vaccines

Historically, the primary approach to specifically activate host T cells against tumor antigens has been therapeutic cancer vaccination. In addition to the successful use of preventative vaccines used in the defense against cancer-causing infectious diseases, including hepatitis B virus and human papillomavirus, the knowledge that patients can harbor CD8+ and CD4+ T cells capable of recognizing tumor expressed antigens hinted at the possibility of developing cancer vaccines [5, 7].

Unfortunately, the general lack of understanding of the mechanisms of immunization, and particularly of the role of dendritic cells (DCs), has led to a series of failures of therapeutic cancer vaccines in initial randomized trials [5, 8]. Early on, it was not appreciated that, by creating an environment that disables the immune response, cancer is able to induce tolerance. Therefore, in contrast to conventional prophylactic vaccines for infectious agents, in order to be effective, cancer vaccination must break the tolerance acquired by the tumor cells [3, 5, 9]. DCs are known to be the most effective antigen presenting cells and play a pivotal role in coordinating innate and adaptive immune responses [10]. Thus, for cancer vaccines to break the tolerance, high quantities of antigens must be targeted to DCs and these, in turn, need to be expanded and activated with appropriate agents [3].

One of the main obstacles to the development of successful cancer vaccines is in identification of the most suitable antigens to use [11]. The earlier vaccine formulations which consisted of short peptides, (usually without an effective DC-activating adjuvant) resulted in minimal clinical effectiveness. This could be attributed to their poor pharmacokinetic properties leading to their rapid clearance before being loaded onto DCs. Without an appropriate activation signal, DCs would probably remain in the steady state and be as likely to induce tolerance as immunity [8]. As it was later shown, the therapeutic efficacy of cancer vaccines can be improved when immune stimulants such as IL-2 are co-administered with short peptide vaccines [12]. However, in some studies, the combination of a cancer vaccine with an immune checkpoint blockade demonstrated no improvement over the blockade alone [13]. Since full-length proteins harbor a wider profile of epitopes that could be presented by DCs, they have also been tested as targets for cancer vaccinations [5]. Preliminary data from a phase II trial that used a recombinant fusion protein encoding a single cancer-testis antigen (melanoma antigen family A3; MAGE-A3) in HLA-A2-positive non-small cell lung cancer (NSCLC) patients, failed to show a statistically significant survival response [14]. However, it should be noted that, although MAGE-A3 expression was assessed in these patients, the level of homogeneity of MAGE-A3 expression was not reported. This is crucial because T cell response would have to diversify to additional cancer antigens in order to evoke immune attack on those subpopulations of lung cancer cells that do not express MAGE-A3 [5]. Whole cells or cell lysates have been exploited as polyvalent sources of tumor antigens [3]. The rationale behind this approach is that a cancer vaccine should contain a wide variety of tumor-associated antigens, thus using cancer cells or their lysate, as the vaccine would overcome the obstacle of antigen selection. However, even GVAX, the most promising vaccine product based on early studies, failed in Phase III trials due to a lack of clinical efficacy. The failure could be attributed to inadequate immunogenicity of the approach and alterations in preparation of the vaccine product required by commercial scale-up [15]. In addition, since cell-based vaccines contain thousands of antigens, they have been criticized for a lack of tumor specificity [3].

**Fig. 1:**
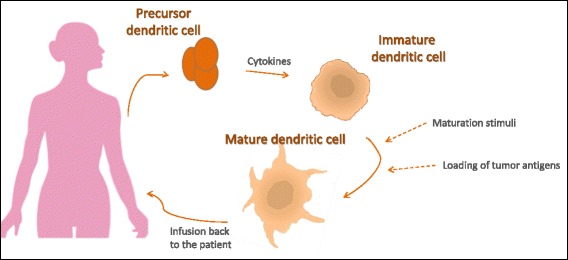
Dendritic cell (DC) based vaccines. CD34+ hematopoietic progenitor cells or monocytes are isolated from the patient’s peripheral blood by cytapheresis. Monocytes are cultured in the presence of Granulocyte macrophage colony-stimulating factor (GM-CSF) and IL-4 to induce differentiation into immature DCs, while CD34+ cells are differentiated when cultured in the presence of GM-CSF, Flt3 ligand and TNF-α. Immature DCs are then loaded with antigen in the form of proteins, peptides or tumor cells either with or following their maturation with proinflammatory cytokines. Once loaded with antigen, DCs can be re-introduced to the patient or frozen in aliquots and thawed before vaccination. (Adapted from [17])

DCs are known as professional antigen presenting cells (APCs), as they are extremely efficient at antigen presentation and induction of T cell immunity when compared with other APCs such as macrophages. These properties have driven attempts to develop DC-based vaccines [10]. In this approach, DCs are isolated from the patient’s peripheral blood mononuclear cells (PBMC), loaded with tumor antigens ex vivo, activated, and then reinfused back into the patient (Fig. 1) [16, 17]. These vaccinations have produced encouraging, albeit modest, clinical results in some patients with advanced cancers. For instance, treatment of metastatic prostate cancer with sipuleucel-T, a cellular product based on enriched blood APCs briefly cultured with a fusion protein consisting of prostatic acid phosphatase linked to the DC growth and differentiation factor granulocyte macrophage colony-stimulating factor (GM-CSF), achieved an approximately 4-month improvement in median survival [18, 19]. The survival benefit of sipuleucel-T ultimately led to US Food and Drug Administration (FDA) approval in 2010 [3]. Despite this increase in survival, randomized clinical trials of sipuleucel-T have failed to show meaningful decreases in tumor volumes or disease response. Furthermore, this approach has not been widely adopted by the biotech-pharmaceutical industry, oncologists, clinical investigators, or patients due to the complications associated with producing and administering the therapy [5].

Obstacles to the success of cancer vaccines administered as “single agents” are still many. The ideal tumor antigens should not just be expressed at high levels in the target tumor population in a significant percentage of patients with a particular cancer type, but should also be expressed at lower levels or not at all in normal tissues to ensure specificity and should be essential for the cancer’s growth or survival in order to minimize the potential for immune escape due to downregulation of antigen expression [20, 21]. Currently, not many antigens fulfill these criteria and even having all of these properties cannot assure the production of a protective T cell response [22–24]. Additionally, it may be inadequate to rely solely on sequencing the expressed tumor genome searching for mutations. Not only can the mutational status and antigen expression within a tumor bed be heterogeneous, but even if expressed, it is not guaranteed that predicted antigenic peptides will be produced and processed as peptide-major histocompatibility complex class I (MHCI) complexes. A few groups have sought to address this issue by coupling bioinformatics and mass spectroscopy of peptides eluted from MHCI molecules from both primary tumors and cell lines [25–27]. This strategy can indeed identify those antigens that yield potential targets, but these peptide-MHCI complexes might still not be capable of triggering potent T cell responses. Further, even if ideal antigens are identified, it remains unclear how best to deliver them to patients.

The ideal vaccine will be one able to trigger the maturation of DCs to a state where they can promote the production of tumor-reactive, CD8+ cytotoxic T cells. It is possible that conditions for immunization will finally be optimized; however, the efficacy of a tumor-specific T cell population may still be compromised by the numerous mechanisms of immunoevasion exploited by tumors to defend against T cell attack. These are not reasons to exclude vaccines from consideration as part of an immunotherapy, but rather to call attention to some of the limitations in assessing success in the absence of other immunological regimes. Work on vaccines should continue in a methodical fashion with human studies, since animal models are unlikely to illuminate the best path forward. In addition, similar to all forms of targeted therapy in cancer, the discovery and application of predictive biomarkers or diagnostics, for the identification of those patients most likely to profit from a given vaccine, will be an important challenge for future development [5, 11].

## Oncolytic virus therapy

OV immunotherapy represents a novel form of cancer therapy that employs native or engineered viruses that selectively replicate in and kill cancer cells [28]. OVs are believed to promote antitumor responses mainly through two distinct mechanisms of action: acute tumor debulking owing to tumor cell infection and lysis and induction/initiation of systemic antitumor immunity [29].

Many of the “hallmarks of cancer”, such as sustained proliferation, usurping cellular apoptotic programs, and inactivating growth suppressors, described by Hanahan and Wineberg [30], favor the selective replication of OVs in malignant cells with minimal toxicity to normal tissues [29]. What has also led to an increased interest in employing viruses for the treatment of cancer is the fact that the viral genome can be modified to augment antitumor activity and attenuate pathogenicity [31]. Some of the numerous modifications that have been developed and tested include the insertion of promoters that restrict the expression of virulence genes to tumor cells or the deletion of pathogenic genes to limit the growth and lytic activity of viruses to cancer cells [32, 33]. Additionally, OVs can be engineered to express specific cytokines that favor immune cell recruitment and activation or to produce T cell co-stimulatory molecules on infected tumor cells, thus facilitating the generation of T cell-activating signals leading to co-stimulation of intratumoral T cells [34–48].

After the viral lysis of tumor cells, tumor associated antigens are released within the vicinity of the tumor, resulting in the induction of mounting, sustained, specific, and often CD8+ T cell-mediated antitumor responses. However, an initial host response to the virus may result in the rapid clearance of the virus before it manages to replicate and infect tumor cells at a magnitude that will ensure the initiation of an efficient vaccination response [28]. Circumvention of this initial response has been achieved using strategies such as PEGylation (covalent conjugation with polyethylene glycol) of the viral coat and polymer coating, which prevent antibody binding and neutralization [49, 50]. Other strategies include the expression of viral gene products, which inhibit antigen presentation, thus preventing recognition by T cells and extending viral infection or the suppression of the host immune system through pretreatment with cyclophosphamide [51, 52].

Numerous viruses have been tested as vectors for OV immunotherapy. Some of them are naturally non-pathogenic to humans, such as Newcastle disease virus (paramyxovirus), reovirus, and Seneca valley virus (picornavirus). Others, including herpes simplex virus, measles virus (paramyxovirus), vaccinia virus (poxvirus), are genetically manipulated to become non-pathogenic [53].

Thus far, the most advanced agent in clinical development is Talimogene laherparepvec (T-VEC), which has recently been approved by the FDA for the treatment of advanced melanoma [54]. T-VEC is a modified oncolytic herpes simplex virus type 1 [34, 55] in which two ICP34.5 genes are deleted to prevent neuronal involvement. These genes have been replaced by the coding sequence for the cytokine GM-CSF [34]. Enhanced local expression and secretion of GM-CSF favors APC recruitment to the tumor microenvironment, thereby promoting the induction of antitumor immunity [34, 35, 56]. Further, ICP47 deletion in T-VEC induces viral replication, enhances antigen presentation, and increases oncolytic therapeutic activity [34, 55].

Following preclinical studies which demonstrated the therapeutic activity of T-VEC in several tumor cell lines [35] and in animal models [34], T-VEC was evaluated in a phase I clinical trial which enrolled patients with a number of different tumor types [36]. The study optimized the virus dose, confirmed good tolerability, and demonstrated evidence of antitumor effect. A phase II multi-institutional study was then conducted in which 50 patients with unresectable stage IIIC or IV melanoma were enrolled [57, 58]. Patients received 10^6^ pfu/mL T-VEC by intratumoral injection as an initial dose and 3 weeks later they were administered 10^8^ pfu/mL every 2 weeks for up to 24 injections. The study demonstrated an objective response rate of 26 % with mild side effects related to fever, fatigue and local injection site reactions.

These findings supported a prospective, randomized clinical phase III trial that enrolled 439 patients with unresectable melanoma (stages IIIb, IIIc, or IV) [59]. This study randomized subjects 2:1 to T-VEC or GM-CSF and aimed for a durable response rate as the primary end point. The study demonstrated a substantially better durable response rate for T-VEC compared with the control arm (16.3 vs. 2.1; *P* < 0.001) and, although the study was not powered for survival, the overall survival was superior in the T-VEC arm. Finally, treatment was well tolerated with only mild side effects, the majority of which were related to fever, fatigue, nausea, and local site reaction. Given these findings, the FDA approved T-VEC to treat advanced melanoma in October 2015 [54]. T-VEC is now the first oncolytic immunotherapy to be approved worldwide and it provides a supplementary option for the treatment of patients with advanced melanoma in addition to the other already approved drugs.

Although promising, there are limitations associated with oncolytic therapy. For instance, immunocompromised patients might not be good candidates because OV-mediated antitumor immunity could be compromised in these patients [28]. Furthermore, while T-VEC is, in comparison with other cancer immunotherapy strategies, a very low toxicity option, there is a limitation regarding the levels of efficiency observed in patients with more advanced disease [28, 29, 31, 57]. For these patients, T-VEC is not likely to be the best option as a monotherapy but its administration combined with cancer immunotherapy could prove particularly effective [28]. The fact that OVs are injected locally into the tumor to avoid pre-existing antiviral immunity is also considered a limitation because, in this case, the virus may not reach tumors in organs that are difficult to reach with an injection [29, 54]. Therefore, and given the disseminated nature of metastatic cancer, it is believed that systemic administration may ultimately be more effective. Despite the restraints, OV therapy has demonstrated a favorable risk-benefit ratio and its approval by the FDA is a considerable milestone in the field [28, 54].

## Adoptive cell therapy

ACT is a promising form of immunotherapy which exploits the antitumor properties of lymphocytes to eradicate primary and metastatic tumor cells [60]. Lymphocytes are firstly isolated from patients’ peripheral blood, tumor-draining lymph nodes or tumor tissue, expanded ex vivo, and reinfused back into the patient [3, 61]. This strategy would, in theory, circumvent the baffling duty of breaking tolerance to tumor antigens and produce a large amount of high avidity effector T cells [5]. Indeed, over the last two to three decades, autologous T cell therapies have demonstrated their potential to induce dramatic clinical responses (and have become a viable therapeutic option) [61, 62].

ACT with tumor-infiltrating lymphocytes (TILs) is an approach where T cells, generally mixtures of CD8+ and CD4+ T cells grown from resected metastatic tumor deposits, are harvested and expanded ex vivo prior to adoptive transfer [61, 63]. This approach attempts to reverse the functional impairment of the tumor-specific T cells that reside within the tumor, and caused by the immune suppressive tumor microenvironment, by growing them prior to the reinfusion in a cocktail of various cytokines [62].

The inclusion of a lymphodepleting conditioning regimen for patients prior to TIL infusion has resulted in durable, complete regression of melanoma [61, 64–66]. Host lymphodepletion is speculated to improve TIL functionality not only by eliminating immunosuppressive cells, such as Treg and myeloid-derived suppressor cells (MDSCs), in the tumor microenvironment but also by increasing levels of homeostatic cytokines IL-7 and IL-15 [67, 68]. In a series of recent clinical trials, [69] 93 patients with metastatic melanoma refractory to standard therapies were infused with autologous TILs in conjunction with IL-2 administration following three different lymphoconditioning regimens. The objective response rates ranged from 49 % to 72 % and the rate increased with a greater degree of lymph depletion. A complete tumor regression was observed in 20 of 93 patients (22 %) and this response was durable, continuing for 37 to 82 months in 19 (95 %) of those 20 patients [69]. Other centers involved in large scale trials (such as the MD Anderson Cancer Centre and the Sheba Medical Centre) have reported consistently high response rates and long-lasting tumor regression following TIL therapy [64].

Despite those encouraging results, ACT with TILs has some obvious disadvantages. Firstly, while lymphodepletion enhances ACT efficacy, especially when ablative radiation therapy is added to the conditioning regimen, it can also be life-threatening and it is still not clear which patients should be considered for this [64]. Other disadvantages include the cost and time required to develop the desired cell populations [70]. Furthermore, application of TIL therapy has been restricted to melanoma. TILs can be isolated from several cancers, however, only those from melanomas consistently carry selective reactivity against the tumors from which they were generated, and melanoma is the only cancer for which TILs have demonstrated clinical activity. It has been suggested that the heightened immunogenicity of melanoma compared with other malignancies is associated with the high frequency of mutational events in this cancer [61].

**Fig. 2:**
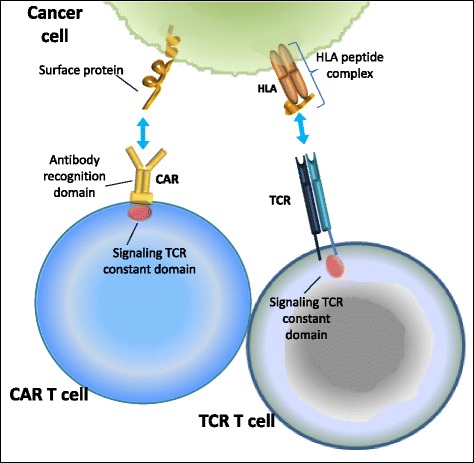
Genetic T cell engineering for the improvement and broadening of tumor-infiltrating lymphocyte (TIL) therapy. Chimeric antigen receptors (CARs) consist of an Ig variable extracellular domain fused to a T cell receptor (TCR) constant domain. The engineered T cells obtain the antigen-recognition properties of antibodies and thus are targeted against any potential cell surface target antigen. The expression of the TCR confers the engineered T cell with the antigen specificity of the transferred TCR. TIL therapy with TCRs is feasible for patients whose tumor harbors the human leukocyte antigen (HLA) allele and expresses the target antigen recognized by the TCR

Ongoing efforts aim not only at improving TIL therapy but also on broadening TIL to battle malignancies. Advances in T cell culturing methods and genetic T cell engineering ensure that clinically relevant numbers of tumor-specific T cells can be generated and delivered as therapy in a timely manner. There are two basic strategies that are being explored in clinical testing of engineered T cells. The first strategy involves the expression of T cell receptor (TCR) α and β chains that confer the engineered T cell with antigen-specificity of the transferred TCR (Fig. 2). This therapy is potentially accessible to any patient whose tumor carries the cognate human leukocyte antigen allele and expresses the target antigen recognized by the TCR. However, the clinical use of highly avid TCRs has been associated with significant secondary destruction of healthy tissues expressing the same target antigen. Ongoing efforts are focused on improving gene transfer efficiencies, designing TCR structural modifications, and identifying target antigens that are highly selective for tumor cells rather than normal cells [71]. Chimeric antigen receptors (CARs) constitute the second approach and consist of an Ig variable domain fused to a TCR constant domain (Fig. 2). The advent of CARs omits the need for tumor cells to carry a functional antigen processing machinery or to express antigens through MHC class molecules since the engineered T cells obtain the antigen-recognition properties of antibodies and are thus potentially targeted against any cell surface target antigen [72].

Tumor regression following administration of genetically engineered cells has been observed in B-cell malignancies, melanoma, and synovial sarcoma, and trials in other types of cancer are ongoing [61]. However, safety issues regarding the selection of the target, the paucity of such targets, serious adverse effects and the lack of long-lasting responses in many patients implies that additional interventions are warranted to appropriately control and activate T cells in the tumor milieu [5].

## Immune checkpoint blockade

Human cancers carry a multitude of somatic gene mutations and epigenetically altered genes, the products of which are potentially recognizable as foreign antigens. Although an endogenous immune response to cancer is observed in preclinical models and patients, this response is not efficient because tumors induce tolerance among tumor-specific T cells and by expressing ligands that bind inhibitory receptors and dampen T cell functions within the tumor microenvironment [3, 5, 73]. One approach to trigger antitumor immune responses has been termed “checkpoint blockade”, referring to the blockade of immune-inhibitory pathways activated by cancer cells [7].

**Fig. 3:**
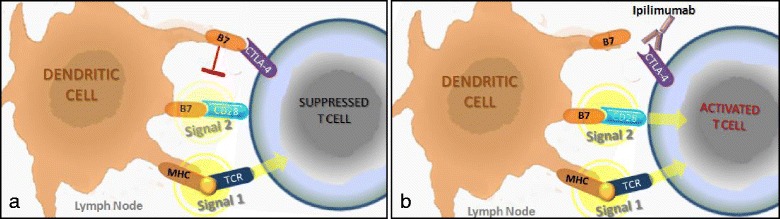
T cell activation in the lymph node. **a** Both immunological signal 1 (T cell receptor (TCR) recognition of antigens) and immunological signal 2 (stimulation of CD28 by B7 costimulatory molecules) are required for T cell activation in the lymph node. The interaction between the cytotoxic T lymphocyte-associated protein 4 (CTLA-4) receptor and B7 expressed on T cells and antigen presenting cells, respectively, prevents T cells from becoming fully activated by blocking immunologic signal 2. **b** Antibodies that block the CTLA-4 pathway (e.g. ipilimumab) permit T cell activation by derepressing signaling by CD28. MCH, Major histocompatibility complex. (Adapted from [77])

CTLA-4, an inhibitory receptor that down-regulates the initial stages of T cell activation (Fig. 3a), was the initial target for checkpoint antibodies [74–76]. The rationale for using anti-CTLA-4 in cancer therapy was to unleash pre-existing anticancer T cell responses (Fig. 3b) and possibly trigger new ones [5, 77]. Antagonist anti-CTLA-4 monoclonal antibodies exhibited antitumor properties in several murine tumor models, such as such as cancers of the ovary, bladder, brain, and fibrosarcoma, while CTLA-4 blockade was ineffective in B16 melanoma, SM1 mammary carcinoma, EL4 lymphoma, M109 lung cancer, and MOPC-315 plasmacytoma models [78]. Ipilimumab, an anti-CTLA-4 antibody, was approved by the FDA in 2011 as a first-line therapy for melanoma patients with metastatic disease, based on phase III trials that showed prolongation of overall survival [4, 13, 79]. Although only a relatively small fraction of patients obtained clinical benefit, these studies clearly establish ipilimumab as an active reagent, offering patients clinically significant benefits and the possibility for long-lasting survival at what is normally the terminal stage of the disease. Additionally, the results validate the idea that activating the T cell compartment can, on its own, provide significant therapeutic benefit [5].

Despite the aforementioned encouraging results, the usage of ipilimumab has shown clinical and scientific challenges. Firstly, as anticipated by the lethal autoimmune phenotype of CTLA-4 knockout mice, grades 3–5 (severe) immune-related adverse events have been observed in 10–35 % of patients undergoing CTLA-4 blockade [80]. The lack of specificity in T cell expansion, coupled with the fundamental importance of CTLA-4 as an immune checkpoint, could account for the significant immune-related toxicities observed in patients treated with ipilimumab [13]. Secondly, in contrast to conventional cytotoxic therapies that directly attack cancer cells and result in a rapid decrease in tumor size, response characteristics with ipilimumab may take several months to manifest, making it difficult to assess response [5]. Nevertheless, ipilimumab has not only provided realistic hope for melanoma patients, especially those with end-stage disease [5], but has initiated a great effort in the search for other immune modulators that can achieve what ipilimumab can, but in a more selective and harmless fashion, with the potential for greater efficiency and frequency of response, and with less autoimmune-related side effects [11].

**Fig. 4:**
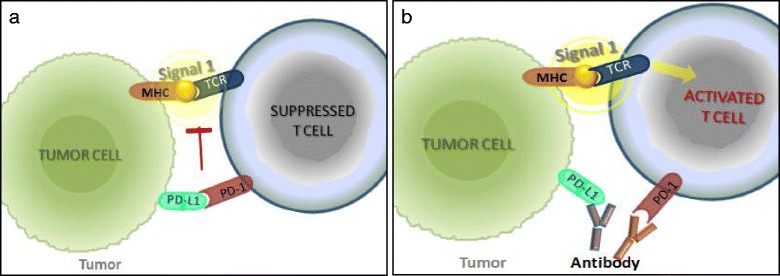
T cell activation in the tumor milieu. **a** Programmed cell death protein 1 (PD1) receptor is an inhibitory receptor expressed by antigen-stimulated T cells. Interactions between PD1 and its ligand, PD-L1, expressed in many tumors activate signaling pathways that inhibit T-cell activity and thus block the antitumor immune response. **b** Antibodies targeting PD1 or PD-L1 block the PD1 pathway and reactivate T cell activity. MCH, Major histocompatibility complex; TCR, T cell receptor. (Adapted from [77])

The downstream signaling of the PD1 receptor, another inhibitory receptor expressed by antigen-stimulated T cells, inhibits T cell proliferation, cytokine release, and cytotoxicity [81–83]. PD1 has two known ligands, PD-L1 and PD-L2 [84, 85]. In tumor models, PD1 signaling inhibits T cells and blocks the antitumor immune response after binding to PD-L1 expressed within the tumor (Fig. 4a) [5]. Inhibition of the interaction between PD1 and PD-L1 (Fig. 4b) can enhance T cell responses in vitro and mediate (preclinical) antitumor activity [86]. Antibodies targeting PD1 or PD-L1 have reached the clinic and include pembrolizumab (previously named as lambrolizumab; anti-PD1) and nivolumab (anti-PD1) [11]. In early phase I trials, PD1-PD-L1 axis blockade alone has yielded promising results in a variety of cancer types; in melanoma, the anti-PD1 antibody nivolumab has shown sufficient clinical responses which are often durable, with some patients remaining free from disease progression for many years [87]. The anti-PD-L1 antibody atezolizumab has induced therapeutic responses in patients within a broad range of human cancers, which included lung, colon, head and neck, and gastric cancers in addition to melanoma and renal cell carcinoma. Thus far, both pembrolizumab and nivolumab have been FDA approved for the treatment of melanoma and NSCLC, while nivolumab has been also approved for the treatment of renal cell carcinoma [4].

These data are consistent with the suggested mechanism of action of this negative regulator. Although CTLA-4 regulates de novo immune responses, the PD1 pathway exerts its major influence on ongoing (effector) immune responses [3]. Particularly, the interaction between PD1 and PD-L1 expressed on activated effector T cells results in inactivation of the PI3 kinase signaling cascade [88, 89] and subsequent blockage of the secretion or production of cytotoxic mediators required for killing. However, it seems that this blockage is rapidly reversible once the inhibition is lifted. Most importantly, the PD-L1 and PD1 antagonists have demonstrated significant response rates and remarkably long-lasting responses [11].

The most striking contrast of the agents that target the PD1-PD-L1 axis to the therapies that block CTLA-4 (ipilimumab) is the favorable toxicity profile of the PD1-PD-L1 blocking agents [90]. The majority of reported cases of toxicity have been readily manageable with supportive care or by immune suppression with steroid administration [11]. The reduced toxicity is consistent with the distinct phenotypes of PD1 genetic knockout mice, which develop delayed-onset organ-specific inflammation as opposed to the uncontrolled global T cell proliferation seen in CTLA-4 knockouts [3], and might hint at the benefits of specifically targeting the properties of cancer that inhibit the immune response rather than non-specific activation of the immune system [11].

Multiple other immune checkpoint pathways that could be the target of novel therapies have been identified. A few examples of those newly discovered molecules that are now being evaluated in preclinical tumor models and/or even in clinical trials are lymphocyte activation gene 3 (LAG3) protein [91] and T cell immunoglobulin and mucin domain-containing 3 (TIM3) protein [92]. From these, therapies targeting LAG3 are the furthest along in clinical development. LAG3 was identified to be progressively expressed on T cells during exhaustion [93] and to be a selective marker of T reg cells, suggesting that it may play a role in immune suppression by tumors. On account of these results, it was speculated that inhibiting LAG3 could enhance antitumor immunity by reversing T cell exhaustion. Agents targeting LAG3, including a fusion protein and LAG3-specific antibodies, have been developed and tested in the clinic either as monotherapy [94] or in combination with anti-PD1 or with conventional therapies [95], demonstrating encouraging results [4]. Human TIM3 is expressed by various T cell populations and by innate immune cells such as DCs [96]. TIM3 coexpression with PD1 on CD8+ tumor infiltrating T cells hinted at the importance of TIM3 in the cancer setting and implied that combination therapies targeting both these pathways are worth exploring [92]. TIM3 antagonists have not been tested in clinical trials but several are in preclinical development [2]. These molecules are just two representatives of the numerous immune checkpoint agents that are currently under development for clinical testing and that are anticipated to improve the antitumor responses when used in combination with other immunologic modalities [4].

## Combination therapies

### Combining immune checkpoint inhibitors

A subgroup of patients with advanced cancers may respond to single-agent immunotherapy, but for the majority, monotherapy may be relatively ineffective [2]. It is thought that, in order to achieve complete remission and cures for patients with cancer, the combination of multiple therapeutic approaches may be required. This field is progressing rapidly to the point that new combinations are being assessed almost monthly [97]. In the following sections, we will mention only a few of the main immunotherapy combinations that have been tested thus far, the successes and failures related to them, and the limitations regarding their administration.

Although both CTLA-4 and PD1 are expressed on T lymphocytes, these negative regulators affect different signaling pathways within these cells; the CTLA-4 checkpoint plays a major role in dampening T cell priming and activation, whereas PD1 blocks effector T cell responses within tissues [3]. Thus, the combination of anti-CTLA-4 and anti-PD1 therapies has been anticipated to demonstrate synergy. Indeed, combination therapy with antibodies targeting both molecules was tested and found to improve antitumor responses in a preclinical animal model [98].

A phase I clinical trial with ipilimumab (anti-CTLA-4) combined with nivolumab (anti-PD1) reported tumor regression in 50 % of treated patients with advanced melanoma [99]. A more recent randomized, placebo-controlled phase II study comparing ipilimumab combined with nivolumab versus ipilimumab alone reported even better responses. Patients with previously untreated metastatic melanoma who received the combination treatment showed an objective response rate of 61 % while, of the patients assigned to the ipilimumab monotherapy, only 11 % demonstrated an objective response [99]. According to a recent, randomized, three-arm phase III clinical trial which compared monotherapy with either ipilimumab or nivolumab to their combination in patients with melanoma, nivolumab alone was less toxic and showed greater clinical benefit than ipilimumab alone [100]. Nivolumab as monotherapy and in combination with ipilimumab demonstrated better objective response rates compared to ipilimumab. From this study the overall survival results are anticipated to shed light on the full effect of combination immunotherapy. On account of these promising efficacy results, there are ongoing clinical trials with anti-CTLA-4 (ipilimumab, Bristol-Myers Squibb or tremelimumab) plus anti-PD1 or anti-PD-L1 in other tumor types such as renal cell carcinoma, NSCLC, small-cell lung, triple-negative breast, pancreatic, gastric, and bladder cancer [97].

Although the combination of immune checkpoint inhibitory antibodies may increase/enhance antitumor immunity, it may also lead to an increase in the magnitude, frequency, and onset of side effects and toxicities (compared with prior experience with either antibody alone) [11]. These side effects resemble autoimmune diseases (such as dermatitis, inflammatory colitis, hepatitis, hypophysitis, and thyroiditis) and, although they can be usually managed with the administration of treatment involving immunosuppression, they clearly identify a requirement for careful dose titrations to define windows of clinical efficacy [3]. Additionally, little is currently known about the long-term effects of combination therapy and whether a different range of immune-mediated toxic effects will manifest with chronic exposure.

### Combination therapy of immune checkpoint inhibitors with conventional therapies

The effects of chemotherapy have always been seen as necessarily harmful to immune mechanisms, however, it is now known that these effects are rather drug-, dose-, and/or schedule-dependent [3]. Conventional cytotoxic treatment regimes, such as chemotherapy, may, in fact, potentiate the antitumor response by releasing multiple tumor neoantigens [101–104]. Chemotherapy may also boost immunotherapies in patients by modifying the immunosuppressive environment of the tumor. Cyclophosphamide is known for depleting Treg cells [105], whereas other chemotherapeutic agents, such as paclitaxel and 5-fluorouracil, eliminate MDSCs [106, 107]. By eliminating the immunosuppressive activities of tumor infiltrating Treg cells and MDSCs, chemotherapy enhances antitumor T cell functions and may lead to more effective inductions of antitumor immune responses [2]. Additionally, combining immune checkpoint inhibitors with chemotherapy may take advantage of the reduction of tumor burden caused by chemotherapy [102]. On the other hand, caution is required when designing clinical protocols because the same agent may prove to be inhibitory, benign, or even stimulatory depending on the phase of immune response being targeted, and even the dose or schedule used [108, 109]. There are chemotherapy regimens that suppress proliferating lymphocytes and could possibly have a negative influence on the effectiveness of immune checkpoint inhibitors that promote the proliferation and activation of TILs. Thus, great care must be taken to use such agents at doses and schedules that do not deplete effector CTLs [5].

This type of combined therapy can lead to an increase in the frequency of adverse events. It has been shown that the combination of ipilimumab with dacarbazine resulted in a survival benefit compared to dacarbazine alone, but this combination therapy had to be discontinued due to synergistic toxicity being observed in several patients [79]. A couple of clinical trials have been completed and many more are ongoing investigating the efficacy and toxicity that can be generated by this combination [97].

**Fig. 5:**
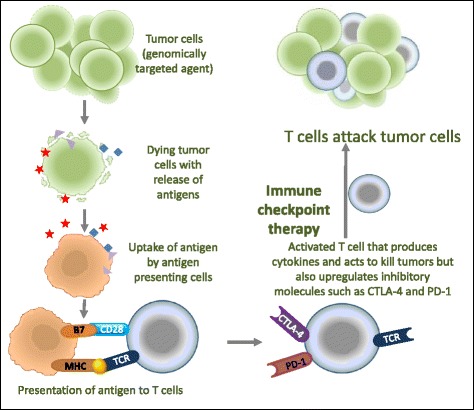
Combination therapy of immune checkpoint inhibitors with conventional therapies may enhance antitumor responses. Molecularly targeted therapies attack cells with specific genetic characteristics resulting in the release of multiple tumor neoantigens. Tumor neoantigens are taken up by antigen presenting cells that then present them in the context of B7 costimulatory molecules and major histocompatibility complex to T cells. T cells are partially activated but overexpress checkpoint molecules, such as CTLA-4 and PD1, which prevent them from becoming fully activated at the tumor site. Immune checkpoint blockade unleashes pre-existing anticancer T cell responses and licenses T cells to attack the cancer cells. CTLA-4, Cytotoxic T lymphocyte-associated protein 4; MCH, Major histocompatibility complex; PD1, Programmed cell death protein 1; PD-L1, PD1 ligand; TCR, T cell receptor. (Adapted from [4])

Similarly to chemotherapy, molecularly targeted therapies expose neoantigens during tumor cell death and they boost/facilitate the antitumor response by priming de novo T cell responses (Fig. 5). The difference is that, while chemotherapy leads to the destruction not only of tumors but also of normal cells, resulting in potential immune response against self-antigens expressed on normal tissues, molecularly targeted therapies, by attacking cancer cells with specific genetic characteristics, restrict the activated immune response generated by immunotherapy agents specifically on tumor antigens. This, in theory, should result in fewer adverse effects [4].

For melanoma patients carrying the V600E activating mutation of B-Raf, treatment with the FDA-approved B-Raf inhibitor vemurafenib has been demonstrated to produce impressive responses in more than half of the patients [110]. However, the development of resistance in some patients requires the administration of a supplementary therapy, which ideally should not simply involve the administration of another inhibitor targeting compensatory pathways (e.g. MEK inhibitor), since the tumor could also easily bypass this strategy. The goal is to generate durable responses and, since immune checkpoint inhibitors have been proven to induce long-term remission, the combination of those two modalities seemed a rational match [5]. Numerous combinations have been attempted with mixed success, and additional combinations are being explored on an ongoing basis. Here are some samples of trials that have already been performed. A phase I clinical trial testing the B-raf inhibitor vemurafenib combined with the anti-CTLA-4 antibody ipilimumab was terminated early due to hepatotoxicity [111]; however, preliminary data from a combination trial of ipilimumab with the BRAF inhibitor dabrafenib indicate that this combination can be tolerable [112]. Nevertheless, triple therapy with ipilimumab, dabrafenib, and trametinib led to colitis in two out of seven patients enrolled in the study. Although the number of patients was small, these cases highlighted the increased possibility of added toxicity with the triple combination over ipilimumab as a single agent and the triple combination cohort of this study was closed [113].

The combination of PD1-PD-L1 axis blockade with B-raf inhibitors alone or in combination with MEK inhibitors is presently being investigated. This approach is based on observations such as the development of resistance to B-Raf inhibitors accompanied by an upregulation of PD-L1 on melanoma cells and the influx of TILs in biopsy samples taken soon after the initiation of BRAF-V600E inhibition in patients with melanoma [114].

Immune checkpoint inhibitors have also been combined with vascular endothelial growth factor (VEGF)-guided therapy. The rationale behind this combination is that, besides promoting angiogenesis, VEGF plays a part in immunity by enhancing the number of Treg cells and MDSCs in the tumor while reducing the intratumoral influx of lymphocytes and suppressing DC maturation. A few studies targeting either the VEGF receptor or VEGF combined with immune checkpoint inhibitors have already been conducted and the results were encouraging [2]. A phase I clinical trial showed that the combination of nivolumab with either sunitinib or pazopanib as a second-line therapy in patients with kidney cancer generated high response rates for both arms [115]. Increased but manageable grade 3–4 toxicities were observed in both combinations, highlighting that further adjustments in dose and schedule may be required to define an optimal regime [97].

According to a study recently published by Hodi et al. [116], the combination of CTLA-4 blockade with ipilimumab and VEGF inhibition with bevacizumab in patients with metastatic melanoma exhibited favorable clinical activity compared with ipilimumab alone, leading to a median survival of 25 months. Side effects included inflammatory events such as giant cell arteritis, hepatitis, and uveitis and, although they were more frequent than anticipated for either drug alone, they were controllable [116]. Bevacizumab combined with the PD-L1-specific monoclonal antibody (mAb) atezolizumab has generated moderate adverse effects in phase I clinical trials [2]. Additional studies have targeted VEGF signaling through neuropilin receptors based on the expression of the latter on a subset of Treg cells and on a specific subgroup of dendritic cells and because neuropilin is upregulated in numerous tumor types with expression being correlated with tumor progression [117–119]. The phase I study of the human mAb MNRP1685A, which targets the VEGF-binding domain of neuropilin1 (NRP1), in patients with advanced solid tumors, showed tolerability [120], while a phase Ib study evaluating an anti-NRP1 mAb, in combination with bevacizumab and paclitaxel in patients with advanced solid tumors, generated a higher than expected proteinuria [121]. This outcome weakens the further testing of anti-NRP1 agent combined with VEGF targeted regimes.

### Combination of immunostimulatory antibodies

Immunostimulatory antibodies represent another class of agents that have been tested either as monotherapy or in combination with the immune checkpoint inhibitors. Most of these target members of the TNF receptor superfamily and, in contrast to immune checkpoint targets such as PD1 or CTLA-4, the goal of most of these antibodies is to activate their target receptors. Thus far, antibodies stimulating the OX40 and 4-1BB receptors are furthest along in clinical development [4]. 4-1BB is an inducible type I membrane glycoprotein expressed on the surface of primed CD4+ and CD8+ T cells. Numerous studies indicate that signaling via 4-1BB either by binding to its ligand or by antibody ligation promotes T cell activation, growth, and survival and enhances effector functions [122].

The significance of the 4-1BB pathway has been highlighted in numerous diseases, including cancer, and it has been previously shown that anti-4-1BB mAbs possess potent antitumor properties derived from their effectiveness in activating and protecting T and NK cells [123]. Urelumab and PF-05082566 are agonistic 4-1BB-specific antibodies that are under evaluation for several malignancies. Although some antitumor activity was observed for urelumab during a phase I trial [124], a phase II trial in patients with melanoma resulted in increased hepatotoxicity leading to therapy discontinuation [2]. Testing of PF-05082566, either as monotherapy or combined with rituximab, exhibited some encouraging results in mixed solid tumors and in non-Hodgkin lymphoma in phase I clinical trials [2].

Interestingly, a recent study showed that tumor-targeting antibodies, such as cetuximab or trastuzumab, induced the upregulation of 4-1BB on NK cells and, when NK cells were stimulated with an agonist 4-1BB antibody, they exhibited an improved cytotoxicity against cancer cells [87]. Thus, preclinical evidence supports the hypothesis that the combination of 4-1BB-specific mAbs with tumor depleting antibodies will show potent synergistic effects [125].

The effects of coupling anti-4-1BB agonist antibodies with immune checkpoint blockade/inhibitors have also been investigated. CTLA-4 inhibition combined with the “trimab” scheme (composed of immune activating anti-CD40 and anti-CD137 mAbs, a blocking antibody against the DR5 receptor for TNR-related apoptosis inducing ligand) substantially increased the tumor rejection rate of established mammary tumors in mice compared to trimab alone [126].

According to more recent studies, combining T cell co-inhibitory blockade with anti-CTLA-4 and active co-stimulation with anti-4-1BB promotes rejection and regression of B16 melanoma and prostate tumors, respectively, in the context of a suitable vaccine [127, 128]. Additionally, agonistic anti-4-1BB antibodies combined with anti-PD1 can enhance the curative capacity of radiotherapy in established breast malignancy [129]. While it is still early, the aforementioned data indicate that the parallel targeting of the immune checkpoint blockade and 4-1BB signaling pathways justifies clinical evaluation [130].

OX40 is a potent costimulatory receptor found primarily on CD4+ and CD8+ T cells and its engagement promotes T cell activation, survival, proliferation, and cytokine production [131, 132]. The natural ligand of OX40 is found on APCs, including DCs, B cells, and macrophages, and also on activated T cells. The expression pattern of those two molecules suggests that the OX40 pathway supports the immune response during T cell activation. Preclinical studies have shown that monotherapy with an OX40 agonist mediated the rejection of various tumors [131–134]. According to the first phase I clinical trial, patients with advanced cancer treated with an agonistic OX40 mAb experienced an acceptable toxicity profile and 12 out of 30 patients showed regression of at least one metastatic lesion [131].

In an attempt to improve the efficacy of OX40 engagement, OX40 agonist antibodies have been paired with chemotherapy, radiotherapy, targeted small-molecule therapeutics, cytokines and adjuvants, immune stimulatory antibodies such as agonist 4-1BB mAbs, and immune checkpoint inhibitors against CTLA-4, PD1, and TIM3. The preclinical data showed that those combined schemes improved tumor rejection, long-term survival, and/or resistance to tumor rechallenge in mice bearing various cancers [2, 130].

Glucocorticoid-induced tumor necrosis factor receptor related gene (GITR) is a costimulatory molecule constitutively expressed on Treg cells. Contrary to Tregs, CD4+ and CD8+ T cells begin to express GITR approximately 24 hours after stimulation, with the expression lasting several days. GITR has also been observed on DCs, monocytes, and NK cells. The GITR ligand is highly expressed on activated APCs and endothelial cells [2, 130]. GITR seems to play a key role in suppressing Treg cell activity, activating proliferation, and in effector functions in CD4+ and CD8+ cells [97]. Preclinical research has demonstrated that activating GITR, by agonist antibodies or natural ligands, can also serve as an effective antitumor therapy [135].

In vitro GITR ligation has previously been shown to augment T cell-mediated antitumor immunity. Cohen et al. [136] were the first to demonstrate that, as a monotherapy, an agonist anti-GITR antibody induced regression of small established B16 melanoma tumors in mice. The GITR agonist was shown to synergize with anti-PD1 therapy to eliminate established tumors [2] and it has also been successfully coupled with other immunotherapies such as DC-based vaccines, adoptive cell transfer, or an antagonistic antibody against CTLA-4 [130]. Although the clinical development of GITR-specific antibodies is limited to date [2], the aforementioned findings provide further support for the continued development of agonistic anti-GITR mAbs as an immunotherapeutic strategy for cancer and antibodies from GITR Inc., Merck, Agenus, and others are in preclinical and early clinical development [2].

Herpes virus entry mediator (HVEM) is another member of the TNFR superfamily widely expressed on APCs, endothelium, and lymphocytes, with the highest expression levels detected on resting T cells [2, 130]. As a molecular switch, HVEM regulates T cell activation in a costimulatory or coinhibitory fashion depending on which ligand it has engaged [137]. HVEM ligands belong to two distinct families: the TNF-related cytokines LIGHT and lymphotoxin-α, and the Ig-related membrane proteins BTLA and CD160 [138, 139]. The binding of LIGHT or lymphotoxin-α to HVEM delivers a stimulatory signal, whereas the binding of BTLA or CD160 to HVEM delivers an inhibitory signal [137].

HVEM and its ligands have been involved in the pathogenesis of various autoimmune and inflammatory diseases, but recent reports indicate that this signaling pathway may also be involved in tumor progression and resistance to immune response [138, 139]. For instance, it has been shown that BTLA weakens antitumor T cell activation by signaling via HVEM [138, 140] and BTLA inhibition augmented the propagation and antitumor activity of melanoma-specific CD8+ T cells [141]. Additionally, BTLA is upregulated in various tumor types, suggesting that this pathway has been appointed for use in immune suppression. Due to these observations, approaches to mono- or combination therapy targeting the HVEM axis have been suggested and therapeutic targeting of the HVEM axis is likely to see clinical development in the near future [2].

The combination of immune checkpoint inhibitors with cancer vaccines was anticipated to elicit a robust response in clinical trials. Although some encouraging results have been reported in mouse models and clinical trials [11, 142], this approach has not yet flourished. What has also been proposed and tested is the combination of immune checkpoint inhibitors with OVs. OVs were initially designed to act as tumor-eliminating therapeutics, but the most recently engineered OVs not only induce immunogenic cell death but also express immune stimulating “cargo” that can be selectively targeted to tumor beds. The coupling of agents that block the immune checkpoint with OVs has been viewed as a natural marriage demonstrated in preclinical models by combining Newcastle disease virus with antibodies against the CTLA-4 receptor [143]. The combination of T-VEC and immune checkpoint blockade has significant preclinical support, it has already been tested in the clinic and large randomized studies are underway. Preliminary data from a phase I trial of T-VEC and ipilimumab did not demonstrate unexpected side effects, while they have reported response rates of 50 % with a 22 % complete response rate, thereby supporting an added therapeutic benefit of combination therapy [144]. Based on these results, a randomized phase II study of ipilimumab plus T-VEC versus ipilimumab alone is ongoing, with target accrual of 200 patients (NCT01740297) [145], while a randomized phase I/II study of pembrolizumab with or without T-VEC is also underway [145].

A myriad of potential combination strategies exist, but immune checkpoint blockade stands out as the backbone of most strategies (Table 2) [13, 100, 146–151]. The main reason is that the immunologic checkpoint inhibitors have continued to show efficacy in a broad variety of tumor types, including those characterized as poorly immunogenic. Nevertheless, while combinational immunotherapies have been quite successful so far, we should not think of them as a panacea. The exact mechanisms for the antitumor effects of these therapies in murine and human tumors remain obscure and, therefore, their combinations may lead to unforeseen consequences.

**Table 2:** Outcomes from key clinical trials of combination immunotherapies (adapted from [97])

Agents	Indication	Regimen or design	n	Overall response (CR and PR)	Survival	Refs
Ipilimumab and nivolumab	Advanced-stage untreated melanoma	Nivolumab or ipilimumab alone versus nivolumab plus ipilimumab	945	-44 % nivolumab -19 % ipilimumab -58 % ipilimumab plus nivolumab	Median PFS: -2.9 months for ipilimumab* -6.9 months for nivolumab^†^ -11.5 months for nivolumab plus ipilimumab*^,†^	[98]
Ipilimumab and nivolumab	Advanced-stage melanoma	Concurrent or sequential combination with dose escalation	53	42 % (concurrent combination)	OS rate: -85 % 1-year -79 % 2-year	[146]
Ipilimumab and nivolumab	Advanced-stage untreated melanoma	Ipilimumab alone versus Ipilimumab plus nivolumab	142	-11 % ipilimumab* -61 % ipilimumab plus nivolumab*	Median PFS: -4.4 months for ipilimumab -Not reached for ipilimumab plus nivolumab	[147]
Ipilimumab and GP100 vaccine	Previously treated advanced-stage melanoma	Ipilimumab or vaccine alone versus ipilimumab plus vaccine	676	-10.9 % ipilimumab alone* -1.5 % vaccine alone^†^ -5.7 % ipilimumab with vaccine*^,†^	Median OS: -10.1 months for ipilimumab alone -6.4 months for vaccine alone* -10.0 months for ipilimumab plus vaccine*	[13]
Ipilimumab and dacarbazine	Advanced-stage untreated melanoma	Dacarbazine alone versus Ipilimumab plus dacarbazine	502	-10.3 % dacarbazine alone -15.2 % ipilimumab with dacarbazine	Median OS: -9.1 months for dacarbazine alone* -11.2 months for ipilimumab plus dacarbazine*	[79]
Ipilimumab and radiotherapy	Post-docetaxel CRPC	Radiotherapy followed by placebo versus radiotherapy followed by ipilimumab	799	NA	Median OS: -10.0 months for radiotherapy followed by placebo -11.2 months for radiotherapy followed by ipilimumab	[149]
Carboplatin plus paclitaxel with placebo or ipilimumab	NSCLC	Placebo control versus phased or concurrent schedule	204	-18 % chemotherapy control -32 % irBORR ipilimumab	Median irPFS: -4.6 months chemotherapy control* -5.7 months for phased ipilimumab*	[150]
Carboplatin plus paclitaxel with placebo or ipilimumab	ED-SCLC	Placebo control versus phased or concurrent schedule	130	-53 % chemotherapy control -71 % irBORR ipilimumab	Median irPFS: -5.3 months chemotherapy control* -6.4 months for phased ipilimumab*	[151]

The difference between pairs of outcomes marked by either * or † reached statistical significance

CR, Complete response; CRPC, Castrate-resistant prostate cancer; ED-SCLC; Extensive-disease small cell lung cancer; irBORR, Immune-related best overall response rate; irPFS, Immune-related progression-free survival; NA, Not available or not presented; NSCLC, Non-small-cell lung cancer; OS, Overall survival; PR, Partial response

Another issue that has also been addressed is the appropriate scheduling of the combined therapies. Some argue that (molecularly) targeted therapies and immunotherapy will not necessarily offer the optimum result in terms of efficacy and safety simply by combining them at the same time. This is supported by the fact that not only do some molecularly targeted therapies also have mmunomodulatory effects, but mutations introduced by some chemotherapies might render subsequent immunotherapies more effective. On the other hand, others argue that it is not clear that a regressing tumor is immunogenic while growing tumors are known to induce inflammatory processes. Thus, cancers may be the most immunogenic while growing and immunotherapy would not be as effective when given after a targeted therapy [152].

What is obvious is that there are still several open questions in cancer immunotherapy as reflected by the empirical rather than rational manner through which the synergistic effects of most of the agents are presently discovered. A more complete understanding of the immune mechanisms of these agents and of the way they interact with the immune system and the tumor itself are warranted to guide the development of combination therapies for clinical trials.

## Biomarkers in cancer immunotherapy

Immunotherapy has been a game-changer in the field of cancer therapy and developments in immune checkpoint-based therapy, in particular, are progressing at a breathtaking pace. Nevertheless, only a fraction of patients respond to these immunotherapies [153]. Therefore, patient selection is an important issue as it will avoid treatment-related toxicity and cost in patients that are unlikely to benefit. This will require the identification and validation of reliable surrogate biomarkers that will provide an early indication of response or predict clinical benefit.

There are several ongoing efforts to identify predictive biomarkers of immune checkpoint therapy (Table 3). Several studies support the hypothesis that immunotherapy is particularly efficient in highly mutagenized tumors [154, 155]. The mutational load is believed to generate neo-antigen-specific T cell responses which are likely to contribute to the clinical responses to immunotherapy. For instance, according to two independent groups, the mutational frequency in melanoma tumors was correlated with clinical responses to anti-CTLA-4 therapy [156]. Similarly, higher numbers of mutations, including mutations in DNA repair pathways, were shown to correlate with clinical responses in patients with colon cancer and NSCLC who were treated with anti-PD1 inhibitors [157, 158]. However, this is not the case for all tumor types since, in different clinical trials, patients with kidney cancer, which has a relatively low mutational frequency, have had noticeable clinical responses to anti-PD1 treatment [159].

**Table 3:** Immunotherapy biomarkers

Biomarker	Comments	Refs
Mutational load	In general, the higher the number of mutations the better the response to immunotherapy; not the case for all tumors	[154–159]
Lymphocyte infiltrates	The presence of lymphocyte infiltrates is related to improved survival	[2, 97]
PD-L1 expression	PD-L1 expression on tumor cells may potentially serve as a useful predictive biomarker for response to anti-PD1/PDL1 therapy; not the case for many tumors	[86, 99, 160]
Genetic profiling	Patients with higher baseline expression of immune-related genes generally respond better to ipilimumab	[97]

PD1, Programmed cell death protein 1; PD-L1, PD1 ligand

For many cancers the presence of lymphocyte infiltrates is related to improved survival [97]. Additionally, it has been suggested that PD-L1 expression on tumor cells may potentially serve as a useful predictive biomarker to identify patients who would benefit from immune checkpoint blockade monotherapy [86]. However, it has been subsequently shown that many patients with PD-L1-negative tumors can still respond to PD1 pathway blockade while some patients with high levels of PD-L1 do not respond [99, 160]. Therefore, the levels of PD-L1 around the tumor microenvironment cannot be considered an optimal biomarker for patient selection and lack of PD-L1 expression cannot be reliably used to exclude patients from treatment with PD1 pathway blockade.

Current studies in several tumors are concentrated on characterizing TILs, including the overexpression of markers of exhaustion such as PD1, LAG3, and TIM3, because, according to studies on murine models [92, 161], the combination of those immune checkpoint inhibitors can be effective in overcoming this exhausted phenotype [2, 92, 161]. What is also understood is that the response to different immunotherapeutic combinations will probably rely on the patient’s immune milieu. Thus, the development of a system that, apart from PD-L1 status and lymphocyte profile, takes into consideration a wider picture of the immune milieu, will be critical in guiding therapeutic combinations. Furthermore, the integration of immunohistochemistry and genetic profiling of the tumor microenvironment could be exploited to classify cancers based on their strategy of immune evasion; this is anticipated to improve biomarker algorithms [2, 97].

## Conclusion

Cancer therapy has long depended on strategies that directly attack tumor cells to treat patients. Cancer immunotherapy, the treatment that harnesses the patient’s immune system to fight cancer, is now emerging as an important addition to conventional therapies. Immune checkpoint blockade therapy, in particular, has undoubtedly been one of the most impressive advancements made in cancer therapeutics in recent years. The impact of this scientific achievement is reflected by the fact that James P. Allison has been recently awarded the 2015 Lasker-DeBakey Clinical Medical Research Award for the discovery and development of an anti-CTLA-4 mAb that releases the brakes of the immune system to combat cancer. Blockade of CTLA-4 with the mAb ipilimumab has already benefited thousands of people with advanced melanoma, a disease that typically used to kill people in less than a year. Most importantly, the clinical success of anti-CTLA-4 created a new field, termed immune checkpoint therapy, and now, not only have additional immune inhibitory checkpoints been released, such as PD1 and its ligand PD-L1, but these are being used in combination with each other or with conventional therapies for the induction of robust and sustained antitumor responses in a wide variety of tumors. While optimal combinations of regimes still need to be determined and extensive efforts must be made in the identification and validation of predictive biomarkers, checkpoint blockade immunotherapy and its combination with other (immune) therapeutic modalities are the leading path to increased therapeutic success across a whole range of tumor types.

## Abbreviations

ACT: adoptive cellular therapy
APCs: antigen presenting cells
CARs: chimeric antigen receptors
CTLA-4: cytotoxic T lymphocyte-associated protein 4
DCs: dendritic cells
GITR: glucocorticoid-induced TNFR-related gene
GM-CSF: granulocyte macrophage colony-stimulating factor
HVEM: herpes virus entry mediator
LAG3: lymphocyte activation gene 3
mAb: monoclonal antibody
MAGE-A3: melanoma antigen family A3
MDSCs: myeloid-derived suppressor cells
MHCI: major histocompatibility complex class I
NRP1: neuropilin1
NSCLC: non-small cell lung cancer
OV: oncolytic virus
PBMC: peripheral blood mononuclear cells
PD1: programmed cell death protein 1
TCR: T cell receptor
TILs: tumor-infiltrating lymphocytes
TIM3: T cell immunoglobulin and mucin domain-containing 3
VEGF: vascular endothelial growth factor
